# Understanding Local Robustness of Deep Neural Networks under Natural Variations

**DOI:** 10.1007/978-3-030-71500-7_16

**Published:** 2021-02-24

**Authors:** Ziyuan Zhong, Yuchi Tian, Baishakhi Ray

**Affiliations:** 8 Universidad Autónoma de Madrid, Madrid, Spain; 9 University of Twente, Enschede, The Netherlands; Columbia University, New York, NY USA

**Keywords:** Deep Neural Networks, Software Testing, Robustness of DNNs

## Abstract

Deep Neural Networks (DNNs) are being deployed in a wide range of settings today, from safety-critical applications like autonomous driving to commercial applications involving image classifications. However, recent research has shown that DNNs can be brittle to even slight variations of the input data. Therefore, rigorous testing of DNNs has gained widespread attention.

While DNN robustness under norm-bound perturbation got significant attention over the past few years, our knowledge is still limited when natural variants of the input images come. These natural variants, e.g., a rotated or a rainy version of the original input, are especially concerning as they can occur naturally in the field without any active adversary and may lead to undesirable consequences. Thus, it is important to identify the inputs whose small variations may lead to erroneous DNN behaviors. The very few studies that looked at DNN’s robustness under natural variants, however, focus on estimating the overall robustness of DNNs across all the test data rather than localizing such error-producing points. This work aims to bridge this gap.

To this end, we study the local per-input robustness properties of the DNNs and leverage those properties to build a white-box (DeepRobust-W) and a black-box (DeepRobust-B) tool to automatically identify the non-robust points. Our evaluation of these methods on three DNN models spanning three widely used image classification datasets shows that they are effective in flagging points of poor robustness. In particular, DeepRobust-W and DeepRobust-B are able to achieve an F1 score of up to 91.4% and 99.1%, respectively. We further show that DeepRobust-W can be applied to a regression problem in a domain beyond image classification. Our evaluation on three self-driving car models demonstrates that DeepRobust-W is effective in identifying points of poor robustness with F1 score up to 78.9%.

## 1 Introduction

Deep Neural Networks (DNNs) have achieved an unprecedented level of performance over the last decade in many sophisticated areas such as image recognition [38], self-driving cars [5] and playing complex games [65]. These advances

(a) 0°, bird (b) +6°, airplane (c) +24°, cat (d) -9°, dog (e) 0°, bird (f) +6°, bird (g) +24°, bird (h) -9°, bird

Fig. 1: **(a)-(d) A well-trained Resnet model [14] misclassifies the rotated variations of a bird image into three different classes though the original un-rotated image is classified correctly. (e)-(h) The same model successfully classifies all the rotated variants of another bird image from the same test set. The sub-captions consist of rotation degrees and the predicted classes.**

have also motivated companies to adapt their software development flows to incorporate AI components [3]. This trend has, in turn, spawned a new area of research within software engineering addressing the quality assurance of DNN components [11, 20, 32, 36, 40, 42, 55, 57, 73, 74, 91, 92].

Notwithstanding the impressive capabilities of DNNs, recent research has shown that DNNs can be easily fooled, i.e., made to mispredict, with a little variation of the input data [14, 23, 73]—either adding a norm-bound pixel-level perturbation into the original input [9, 23, 71], or with *natural* variants of the inputs, e.g., rotating an image, changing the lighting conditions, adding fog etc. [14, 52, 55]. The natural variants are especially concerning as they can occur naturally in the field without any active adversary and may lead to serious consequences [73, 92].

While norm-bound perturbation based DNN robustness is relatively well-studied, our knowledge of DNN robustness under the natural variations is still limited—we do not know which images are more robust than others, what their characteristics are, etc. For example, consider Figure 1: although the original bird image (a) is predicted correctly by a DNN, its rotated variations in images (b)-(d) are mispredicted to three different classes. This makes the original image (a) very weak as far as robustness is concerned. In contrast, the bird image (e) and all its rotated versions (generated by the same degrees of rotation) in Figure 1:(f)-(h) are correctly classified. Thus, the original image (e) is quite robust. It is important to distinguish between such robust vs. non-robust images, as the non-robust ones can induce errors with slight natural variations.

Existing literature, however, focuses on estimating the overall robustness of DNNs across all the test data [4, 14, 88]. From a traditional software point of view, this is analogous to estimating how buggy a software is without actually localizing the bugs. Our current work tries to bridge this gap by localizing the non-robust points in the input space that pose significant threats to a DNN model's robustness. However, unlike traditional software where bug localization is performed in program space, we identify the non-robust inputs in the data space. As a DNN is a combination of data and architecture, and the architecture is largely uninterpretable, we restrict our study of non-robustess to the input space. To this end, we first quantify the local (per input) robustness property of a DNN. First, we treat all the natural variants of an input image as its *neighbors*. Then, for each input data, we consider a population of its neighbors and measure the fraction of this population classified correctly by the DNN - a high fraction of correct classifications indicates good robustness (Figure 1:e) and vice versa (Figure 1:a). We term this measure *neighbor accuracy*. Using this metric, we study different local robustness properties of the DNNs and analyze how the weak, *a.k.a.* non-robust, points differ characteristically from their robust counterparts. Given that the number of natural neighbors of an image can be potentially infinite, first we performed a more controlled analysis by keeping the natural variants limited to spatially transformed images generated by rotation and translation, following the previous work [4, 14, 88]. Such controlled experiments help us to explore different robustness properties while systematically varying transformation parameters.

Our analysis with three well-known object recognition datasets across three popular DNN models, i.e., a total of nine DNN-dataset combinations, reveal several interesting properties of local robustness of a DNN *w.r.t.* natural variants:

– The neighbors of a weaker point are not necessarily classified to one single incorrect class. In fact, the weaker the point is its neighbors (mis)classifications become more diverse.
– The weak points are concentrated towards the class decision boundaries of the DNN in the feature space.

Based on these findings, we further develop two techniques (a black-box and a white-box) that can localize the points of poor robustness, thereby providing a means of, input-specific, real-time feedback about robustness to the end-user. Our white-box and black-box detectors can identify weak, *a.k.a.* non-robust, points with f1 score up to 91.4% and 99.1%, respectively, at neighbor accuracy cutoff 0.75. To further check the generalizability of our technique, we aim to detect weak points *w.r.t.* a self-driving car application where we generated natural input variants by adding rain and fog. Note that these are more complex image transformations, and also the model works in a regression setting instead of classification. These models take an image as input, and output a driving angle. Our white-box detector can identify weak points with f1 score up to 78.9%.

In summary, we make the following contributions:

– We conduct an empirical study to understand the local robustness properties of DNNs under natural variations.
– We develop a white-box (DeepRobust-W) and a black-box (DeepRobust-B) method to automatically detect weak points.
– We present a detailed evaluation of our methods on three DNN models across three image classification datasets. To check the generalizability of our findings, we further evaluate DeepRobust-W in a setting with non-spatial transformations (i.e., rain and fog), a different task (i.e., regression), and a safety-critical application (i.e., self-driving car). We find that DeepRobust can successfully detect weak points with reasonably good precision and recall.
– We made our code public at https://github.com/AIasd/DeepRobust.

## 2 Background: DNN Testing

Existing studies have proposed different techniques to generate test data inputs by perturbing input images for a DNN and use them to evaluate the robustness of the DNN. Depending on how the input image is perturbed, the techniques for generating DNN test data can be classified into three broad categories:

*i) Adversarial inputs* are typically generated by norm-based perturbation techniques [9, 23, 39, 46, 53, 85] where some pixels of an input image ($I$) are perturbed by norm-based distance ($l_1$,$l_2$ or $l_{\text{inf}}$) such that the distance between the perturbed image and $I$ is $\leq \epsilon$, where $\epsilon$ is a small positive value. These adversarial examples are used to expose the security vulnerabilities of DNNs.

*ii) Natural variations* are generated through a variety of image transformations, and are used to evaluate the robustness of DNNs under such variations [13, 14, 73]. Sources of these variations include changes in camera configuration, or variations in background or ambient conditions. The transformations simulating these variations could be spatial, such as rotation, translations, mirroring, shear, and scaling on images, or non-spatial transformations, such as changes in the brightness or contrast of an image. Here we first focus on spatial transformations as opposed to adversarial one for two reasons. First, compared with adversarial examples, which is fairly contrived, spatial transformations are more likely to arise in more benign environments. Second, using simple parametric spatial transformations like rotations and translations, it is easier to systematically explore the local robustness properties. Later, to emulate a more natural variation we add fog and rain on the images of self-driving car dataset and evaluate our method's generalizibility.

*iii) GAN-based* image generation techniques use Generative Adversarial Network (GAN) to synthesize images. GAN is one class of generative models trained as a minimax two-player game between a generative model and a discriminative model [22]. GAN-based image generation has been successfully used to generate DNN test data instances [92, 93].

**Standard Accuracy vs. Robust Accuracy.** Standard accuracy measures how accurately an ML model predicts the correct classes of the instances in a given test dataset. Robust, *a.k.a.* adversarial accuracy, estimates how accurately an ML model classifies the generated variants [76]. In this paper, we adopt a point-wise robust accuracy measure, *neighbor accuracy*, to quantify the robustness of a DNN for the neighbors around each data point.

## 3 Methodology

### 3.1 Terminology

**Original Data Point:** An original data point represents an original un-modified data instance (image in our case) in the studied dataset. The original data points can come from training, validation, or testing dataset, depending on the experimental setting. In Figure 2, the triangle in the center is an original data point.

**Neighbors:** Neighbors are images generated by the natural variations, e.g., spatial transformations applied to an original image. Since the transformation parameters are continuous (e.g., degree of rotations), there can be an infinite number of neighbors per image. In Figure 2, the small circles around an original data point represent its neighbors.

**Neighbor Accuracy:** We define *neighbor accuracy* as the percentage of its neighbors, including itself, that can be correctly classified by the DNN model. Figure 2 illustrates this; here, red small circles indicate misclassified neighbors, while the green small circles are correctly classified ones. The figure shows that there are only five neighbors per original data point. In the left-hand-side diagram, four out of five neighbors are correctly classified by the given DNN model. If the original data point is correctly classified as well, the neighbor accuracy of the original data is (5/6=) 83.3%. Similarly, in Figure 2 (right), four out of the five neighbors have been misclassified by the model; if the original data point is misclassified, the neighbor accuracy is (1/6=) 16.6%.

**Robustness.** An original data point is strong, *a.k.a.* robust, w.r.t. the DNN model under test if its neighbor accuracy is higher than a pre-defined threshold. Conversely, a weak, *a.k.a.* non-robust, point has the neighbor accuracy lower than a pre-defined threshold. For example, at 0.75 neighbor accuracy threshold, the black triangle in Figure 2 is a strong point, and the grey triangle is a weak point.

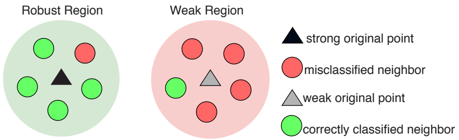

Fig. 2: **Illustrating our terminologies. The triangles are original points, and the small circles are their neighbors generated by natural variations. The light-green region is robust with higher neighbor accuracy, while the light-red region is vulnerable. The corresponding original points are robust and non-robust accordingly.**

A region contains an original point and all of its neighbors. If the original point is strong (weak), we call the corresponding region as a robust (weak) region. In Figure 2, the light green region is robust while the light red region is weak.

**Neighbor Diversity:** For multi-class classification task, different neighbors of an original point can be mis-classified to different classes. Neighbor Diversity score measures how many diverse classes a point's neighbors are classified, and is formally computed using Simpson Diversity Index ($\lambda$) [67]: $\lambda = \sum_{i=1}^{k} p_i^2$ (1)

where $k$ is the total number of possible classes and $p_i$ is the probability of an image's neighbors being predicted to be class $i$. Large Simpson Index means low diversity. Let's consider we have three possible classes A, B, and C. Assume an image has 4 neighbors. Including the original image, there are 5 images in total. If two of the five images are classified as A, and rest are classified as B, then $\lambda = (2/5)^2 + (3/5)^2 + (0/5)^2 = 0.52$. In contrast, if two of them are classified as A, and two are classified as B, and one is classified as C then $\lambda = (2/5)^2 + (2/5)^2 + (1/5)^2 = 0.36$. Clearly, the latter case is more diverse and thus, has a lower $\lambda$ score.

**Feature Representation:** In a DNN, the neurons' output in each layer capture different abstract representation of the raw input, which are commonly known as features, extracted by the current layer and all the preceding layers. Each layer's output forms the corresponding feature space. For a given input data point, we consider the output of the DNN's second-to-last layer as its feature representation or feature vector.

## 3.2 Data Collection

**Neighbor Generation:** For the image classification tasks, for each original image point, we generate its neighbors by combining two types of spatial transformations: rotation and translation. We carefully choose these two types as representatives of non-linear and linear spatial transformations, respectively, following Engstrom et al. [14]. In particular, following them, we generate a neighbor by randomly rotating the original point by t ($\in [-30, 30]$) degrees, shifting it by $dx$ (about 10% of the original image's width i.e. $\in [-3, 3]$) pixels horizontally, and shifting it by $dy$ (about 10% of the original image's height i.e. $\in [-3, 3]$) pixels vertically. It should be noted that for image classification it is standard in the literatures [14, 15, 86] to assume that the transformed image has the same label as the original one. As the transformation parameters are continuous, there can be infinite neighbors of an original data point. Hence, we sample $m$ neighbors for each original data point. We explore the impact of $m$ in RQ2.

For the self-driving-car task where the model predicts steering angle, for each original image point, we generate 50% neighbors with rain effect and the rest 50% with fog effects. We adopt a widely used self-driving car data augmentation package, Automold [60], for adding these effects where we randomly vary the degrees of the added effect. For the rain effect, we set "rain_type=heavy" and everything else as default. For the fog effect, we set everything as default.

**Estimating Neighbor Accuracy:** To compute the neighbor accuracy of a data point for a given DNN model, we first generate its neighbor samples by applying different transformations—spatial for image classification and rain or fog for self-driving-car application. Then we feed these generated neighbors into the DNN model and compute the accuracy by comparing the DNN's output with the label of the original data point. For self-driving-car application, we follow the technique described in DeepTest [73]. More specifically, if the predicted steering angle of the transformed image is within a threshold to the original image, we consider it as correct. This ensures that any small variations of steering angle are tolerated in the predicted results. We then compute $neighbour\ accuracy = \frac{\#correct\ predictions}{original\ point + \#total\ neighbours}$.

## 3.3 Classifying Robust vs. Weak Points

We propose two methods, DeepRobust-W and DeepRobust-B, to identify whether an unlabeled input is strong or weak *w.r.t.* a DNN in real time. If a test image is identified as a weak point, although it may be classified correctly by the pre-trained model, this image is in a vulnerable region where a slight change to this image may cause the pre-trained DNN to misclassify the changed input.

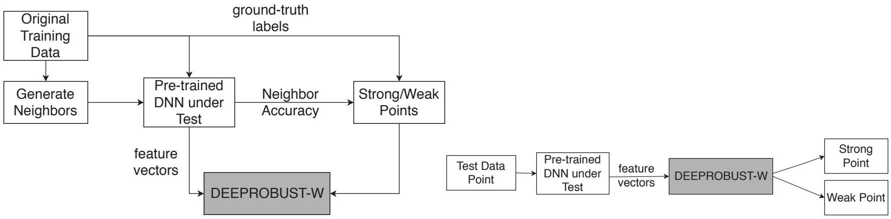

(a) workflow - training (b) workflow - testing

Fig. 3: **Workflow of DeepRobust-W**

**DeepRobust-W: White-box Classifier** This is a binary classifier designed to classify an image (in particular, image feature vector) as a strong or weak point. Here, we assume that we have white box access to the DNN under test to extract the feature vectors of the input images from the DNN. These feature vectors are given as inputs to DeepRobust-W. Figure 3 shows the workflow.

*Training*: During training of DeepRobust-W, we first feed all the original training images and their neighbors to the DNN under test. From the DNN outputs, we compute the neighbor accuracy for each data point in the training set and label each point strong/weak depending on whether its neighbor accuracy is higher/lower than a predefined threshold. For each original data point, we also extract the output of the DNN's second-to-last layer as its feature vector. We use these vectors as inputs to train DeepRobust-W and the outputs are the corresponding strong/weak labels.

*Testing*: Given a test input, we extract its feature vector by feeding the test image to the DNN under test and then feed the extracted feature vector to the trained DeepRobust-W, which predicts if the input is a strong or weak point.

**DeepRobust-B: Black-box Classifier** This is also a binary classifier that is intended to classify an image to strong/weak point. However, here the user does not have white box access to the DNN under test. Figure 4 shows the workflow.

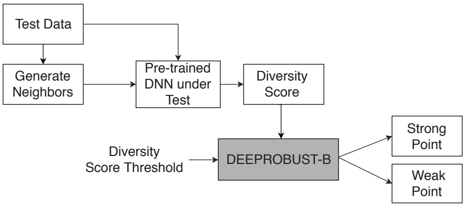

Fig. 4: **Workflow of DeepRobust-B**

Given a test input, we first randomly generate some of its neighbors. We then query the DNN under test with all these neighbors and compute the diversity score, as per Equation 1. If the neighbor diversity score (inversely correlated with neighbor diversity) is greater than a given diversity score threshold, the given test input is classified as a strong point; otherwise, a weak point.

Notice that, in this method, we do not need a training step. We only need the diversity score threshold, which can be empirically set using a ground-truth data set. In particular, we first calculate the neighbor accuracy and diversity score of each pre-annotated point. Next, based on a given neighbor accuracy threshold, we identify the weak points, as the ground truth. The highest diversity score among these weak points is chosen as the diversity score threshold.

**Usage Scenario** DeepRobust-W/B works in a real-world setting where a customer/user runs a pre-trained DNN model in real-time which constantly receives inputs and wants to test if the prediction of the DNN on a given input can be trusted. DeepRobust-W assumes that the user has white-box access to DNN under test and all the training data used to train the DNN. DeepRobust-W leverages the feature vector and neighbor accuracy of the training data to train the classifier, which can notify the user if the current input is a strong point or weak point. If the input is classified as strong point, the user can give more trust to the original DNN's prediction. On the other hand, if the point is classified as a weak point, the user may want to be more cautious about the DNN's prediction and conduct additional inspections.

In the blackbox setting, DeepRobust-B assumes the user does not have white-box access to DNN under test. DeepRobust-B comes with a small overhead of transforming the input multiple times to get some neighbors and querying DNN under test on them to estimate the diversity score.

## 4 Experimental Design

### 4.1 Study Subjects

**Image Classification** Similar to many existing works [36,41,61,73,74,92] on DNN testing, in this work, we use image classification application of DNNs as the basis of our investigation. This is one of the most popular computer vision tasks, where the model tries to classify the objects in an image or video.

**Datasets:** We conduct our experiments on three image classification datasets: F-MNIST [87], CIFAR-10 [37], and SVHN [89].

- **CIFAR-10**: consists of 50,000 training and 10,000 testing 32x32 color images. Each image is one of ten digit classes.
- **F-MNIST**: consists of 60,000 training images and 10,000 testing 28x28 gray-scale images. Each image is one of ten fashion product related classes.
- **SVHN**: consists of 73,257 training images and 26,032 testing images. Each image is a 32x32 color cropped image of house numbers collected from Google Street View images.

**Architectures:** The popular DNN-based image classifiers are variants of convolutional neural networks (CNN) [28,38,79]. Here we study the following three architectures for all the three datasets:

- **ResN**: Following Engstrom *et al.* [14], we use ResN model with 4 groups of residual layers with filter sizes 16, 16, 32, and 64, and 5 residual units each.
- **VGG**: We use the same VGG architecture as proposed in [66].

– **WRN**: We use a structure with block type (3, 3) and depth 28 in [90] but replace the widening factor 10 with 2 for less parameters and faster training.

We train all the models from scratch using widely used hyper-parameters and achieve accepted level of validation natural accuracy). When training models on CIFAR-10, we pre-process the input images with random augmentation (random translation with $dx, dy \in [-2, 2]$ pixels both horizontally and vertically) which is a widely used preprocessing step for this dataset. When training models on the other two datasets, plain images are directly fed into the models. The natural accuracies and robust accuracies of the models are shown in Table 1.

Table 1: **Study Subjects (values are in percentage)**

| Dataset | CIFAR-10 | | | SVHN | | | F-MNIST | | |
|---|---|---|---|---|---|---|---|---|---|
| Model | VGG | ResN | WRN | VGG | ResN | WRN | VGG | ResN | WRN |
| nat acc' | 89.0 | 89.3 | 90.6 | 94.5 | 95.3 | 95.2 | 93.4 | 93.5 | 93.6 |
| rob acc* | 75.5 | 68.5 | 74.8 | 78.1 | 78.9 | 81. | 61.1 | 63.0 | 64.2 |

'Natural accuracy. *Robust accuracy is estimated as the average neighbor accuracy for test data points.

**Steering Angle Prediction** We further evaluate DeepRobust-W in a self-driving car application to show that it can be applied into a regression task. These models learn to steer (i.e., predict steering angle) by taking in visual inputs from car-mounted cameras that record the driving scene, paired with the steering angles from a human driver.

**Datasets:** We use the dataset by Stocco *et al.* [68], which is collected by the authors driving on three tracks of different environments in the Udacity Simulator [77]. It consists of 37888 central camera training images and 9427 central camera evaluation images. Each image is of size 320x120.

**Architectures:** We evaluate our method on the three pre-trained DNN models used in [68]: NVIDIA DAVE-2 [6], Epoch [2], and Chauffeur [1]. These models have been used by many previous testing works on self-driving car [55,68,73].

### 4.2 Evaluation

**Evaluation Metric.** We evaluate both DeepRobust-W and DeepRobust-B for detecting weak points under twelve and nine different DNN-dataset combinations, respectively, in terms of precision, recall, and F1 score. Let us assume that $E$ is the number of weak points detected by our tool and $A$ is the the number of true weak points in the ground truth set. Then the precision and recall are $\frac{|A \cap E|}{|E|}$ and $\frac{|A \cap E|}{|A|}$, respectively. F1 score is a single accuracy measure that considers both precision and recall, and defined as $\frac{2 \times precision \times recall}{precision + recall}$. We perform each experiment for two thresholds of neighbor accuracy that defines strong vs. weak points: 0.75 and 0.50.

**Baselines.** We compare DeepRobust-W and DeepRobust-B with two baselines. One naive baseline (denoted *random*) is randomly selecting the same number of points as detected by our proposed method to be weak points. Another baseline (denoted *top1*) is based on prediction confidence score—if the confidence of a data point is higher than a pre-defined cutoff we call it a strong point, weak otherwise. This baseline is based on the intuition that DNNs might not be confident enough to predict the weak points.

## 5 Results

In this section, we elaborate on our results. In our preliminary experiments, we have two findings regarding neighbor accuracy. First, the neighbor accuracy vary widely across data points and there is a non-trivial number of points having relatively low neighbor accuracy. For example, for all the models trained on CIFAR-10 dataset, 40% of training data and 42% of testing data have neighbor accuracy <0.75, and 16% of training data and 20% of testing data have neighbor accuracy <0.50. These points degrade the aggregated spatial robustness of the model. The same finding holds for the other two datasets. Second, the distribution of neighbor accuracy for a dataset is similar across different models. For CIFAR-10, F-MNIST and SVHN, 60%, 76%, and 81%, respectively, of data points have neighbor accuracy change $< 0.2$ across any two models on the same dataset. This implies that a large portion of data points' neighbor accuracy is independent of the model selected.

The first observation shows that neighbor accuracy is a distinguishable measure for local robustness for the datasets and models we study. The second observation implies that the properties of points of low neighbor accuracy may be similar across models for each dataset. Following these two observations, we dive deeper and explore the characteristics of data points with different neighbor accuracy in RQ1. We then evaluate the performance of DeepRobust-W and DeepRobust-B which are developed based on the observations from RQ1 in RQ2 and RQ3, respectively. Finally, in RQ4, we evaluate the generalizability of our method by applying DeepRobust-W in a regression task for self-driving cars under more complex transformations.

### RQ1. What are the characteristics of the weak points?

We explore the characteristics of robust vs. non-robust points in their feature space. In particular, we check the difference in feature representations between: a) robust and non-robust points, and b) points with different degrees of robustness.

RQ1a. Given a well trained model, do the feature representations of robust and non-robust points vary? In this RQ, we first explore how robust (i.e., strong) and non-robust (i.e., weak) data points are distributed in the feature space.

We apply t-SNE[44], a widely used visualization method, to visualize the distribution of points of different neighbor accuracy in the representation space for all three datasets when using ResN as the classifier. Figure 5 shows the visualization of feature vectors from two randomly picked classes with colors indicating the neighbor accuracy of each point. The darker a point's color is, the lower its neighbor accuracy is. It is evident that most points of low neighbor accuracy tend to be further away from the class center.

To numerically verify this observation, first, we define a class center $c_k$ for each class $k$ as the median value of the feature vectors of all the points from class $k$. Thus, if $f_i$ is the feature of a point at i$^{th}$ dimension and $\hat{f_{ik}}$ is the median of the i$^{th}$ dimension features for all the points in class $k$, $c_k$ is defined to be $(\hat{f_{1k}}, ..., \hat{f_{jk}}, ..., \hat{f_{nk}})$.

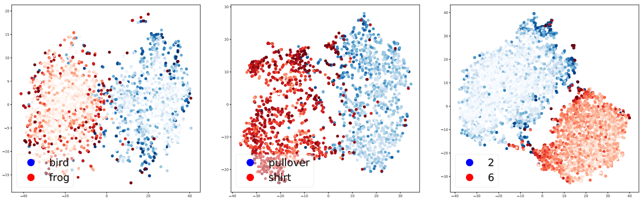

(a) CIFAR-10 (b) F-MNIST (c) SVHN

Fig. 5: **The t-SNE plots of data points from two randomly chosen classes across three datasets using ResNet. Darker color indicates lower neighbor accuracy.**

The reason we take median rather than mean is that it is a more statistically stable measure and is less likely to be heavily influenced by outliers in the representation space. Then, for every point $p$, we define a ratio: $r^{(p)} = \frac{d^{(p)}_{same\_class}}{d^{(p)}_{nearest\_other\_class}}$, where $d^{(p)}_{same\_class}$ is the distance of the $p$-th point's feature vector to its own class center and $d^{(p)}_{nearest\_other\_class}$ is the distance of the $p$-th point's feature vector to the class center of its closest other class. A small $r^{(p)}$ means that the point $p$ is close to its own class center while far from other classes, i.e., $p$ is far from the decision boundary. In contrast, a larger $r^{(p)}$ indicates that the point $p$ is closer to some other classes, i.e., it is closer to the decision boundary.

We then measure the average $r^{(p)}$ among the weak points (denoted as $r_w$) and among strong points (denoted as $r_s$) for all three datasets across three models. Besides, we also calculate mann-whitney wilocox test[47] and cohen's d effect size [10] between the two ratios to test if the two ratios indeed have statistically significant difference and how large the difference is.

Table 2: **Weak and strong points ratio, and cohen's d effect size**

| Dataset | CIFAR-10 | | | SVHN | | | F-MNIST | | |
|---|---|---|---|---|---|---|---|---|---|
| Model | ResN | WRN | VGG | ResN | WRN | VGG | ResN | WRN | VGG |
| Neighbor Accuracy Cutoff=0.5 | | | | | | | | | |
| $r_w$ | 0.915 | 0.955 | 1.004 | 1.046 | 1.103 | 0.997 | 0.746 | 0.734 | 0.976 |
| $r_s$ | 0.609 | 0.584 | 0.975 | 0.294 | 0.309 | 0.977 | 0.297 | 0.293 | 0.930 |
| d* | 1.368 | 1.736 | 1.163 | 2.077 | 2.428 | 1.420 | 1.426 | 1.312 | 1.332 |
| Neighbor Accuracy Cutoff=0.75 | | | | | | | | | |
| $r_w$ | 0.778 | 0.796 | 0.992 | 0.604 | 0.671 | 0.983 | 0.516 | 0.496 | 0.953 |
| $r_s$ | 0.588 | 0.558 | 0.973 | 0.260 | 0.274 | 0.977 | 0.253 | 0.257 | 0.918 |
| d* | 0.786 | 1.040 | 0.749 | 0.860 | 1.111 | 0.401 | 0.749 | 0.642 | 0.937 |

*Cohen's d effect size of 0.20 = small, 0.50 = medium, 0.80 = large, 1.20 = very large, and 2.0 = huge [10, 59].

As shown in Table 2, for both the neighbor accuracy cutoff (0.5 and 0.75), except one setting, the cohen's d effect size for every setting is larger than 0.50, which implies a medium to very large difference. Besides, for every setting, the mann-whitney wilocox test value (not shown in the table) is smaller than $1e^{-80}$, which implies the difference is indeed statistically significant.

The visualization and numerical results imply that most weak points are close to the decision boundaries between classes. Note that similar observation was also observed by Kim et. al. [36] in case of adversarial perturbation. In particular, they find that adversarial examples tend to be closer to class decision boundaries. In contrast, we focus on spatial robustness and find that spatially non-robust points are closer to decision boundaries.

RQ1b. Given a well trained model, do the feature representations of the data points vary by their degree of robustness? By analyzing the classifications of the neighbors of weak vs. strong points, we observe that the weaker a point is, its neighbors are more likely to be classified in different classes. We quantify this observation by computing diversity of the outputs a point's neighbor; We adopt Simpson Diversity Index ($\lambda$) [67] as defined in Equation (1).

Table 3 shows the Spearman correlation between neighbor accuracy and $\lambda$ on the three datasets and three models for each. Note that while calculating the correlation, we remove points with neighbor accuracy 100% since there are many points having 100% neighbor accuracy and tend to bias upward the Spearman Correlation; if we include points with neighbor accuracy 100%, the correlations become even higher. We notice that for any setting, the Spearman Correlation is never lower than 0.853. This indicates that neighbor accuracy and diversity are highly correlated with each other. For example, the bird image in Fig.1a has neighbor accuracy 0.49 and diversity 0.36, while the bird image in Fig.1e has neighbor accuracy 1 and diversity 1. This shows, the classifier tends to be confused about weak points and mispredicts them into many different kinds of classes.

Table 3: **Spearman Correlation between Neighbor Accuracy and Simpson Diversity Index. All coefficients are reported with statistical significance ($p < 0.05$).**

| Dataset | CIFAR-10 | | | SVHN | | | F-MNIST | | |
|---|---|---|---|---|---|---|---|---|---|
| Model | ResN | WRN | VGG | ResN | WRN | VGG | ResN | WRN | VGG |
| corr.coeff. | 0.853 | 0.909 | 0.946 | 0.970 | 0.984 | 0.983 | 0.923 | 0.962 | 0.8947 |

**Result 1:** *In the representation space, weak points tend to lie towards the class decision boundary while the strong points lie towards the center. The weaker an image is, the model tends to be more confused by it, and classify its neighbors into more diverse classes.*

## RQ2. Can we detect the weak points in a white-box setting?

We explore this RQ using DeepRobust-W, as discussed in Section 3.3. DeepRobust-W takes the feature vector of a data point as input and classifies it to a strong/weak point. We implement DeepRobust-W with a simple 4-layer, fully connected neural network architecture with hidden layer dimensions 1500, 1000, and 500, respectively.

Table 4 shows the result. At 0.75 setting, DeepRobust-W has F1 up to 91.4%, with an average of 76.9%. At 0.50 setting, DeepRobust-W detects weak points with average F1 of 61.1%, while it can go up to 79.1%. DeepRobust-W consistently performs significantly better than the baseline methods.

The top1 has very good precision, since a mis-classified image with low confidence tends to have very poor local robustness. However, there also exist many images that are correctly classified with high confidence yet have poor local robustness. The miss of these points leads the top1 to have very poor recall and thus even worse F1 compared with the random baseline. Our method comes to aid by providing high recall at the same time of decent precision.

Table 4: **Performance of DeepRobust-W and the baseline methods for predicting weak points.**

| dataset | model | method | 0.75 neighbor acc. | | | 0.50 neighbor acc. | | |
|---|---|---|---|---|---|---|---|---|
| | | | f1 | tp | fp | f1 | tp | fp |
| CIFAR-10 | ResN | ours | 0.79 | 3844 | 764 | 0.581 | 1290 | 664 |
| | | top1 | 0.376 | 1218 | 206 | 0.182 | 255 | 120 |
| | | random | 0.488 | 2372 | 2236 | 0.233 | 520 | 1445 |
| | WRN | ours | 0.747 | 2901 | 906 | 0.56 | 947 | 610 |
| | | top1 | 0.35 | 889 | 222 | 0.183 | 189 | 90 |
| | | random | 0.395 | 1534 | 2273 | 0.154 | 261 | 1296 |
| | VGG | ours | 0.654 | 2222 | 938 | 0.493 | 747 | 543 |
| | | top1 | 0.439 | 1070 | 153 | 0.266 | 278 | 106 |
| | | random | 0.332 | 1127 | 2033 | 0.132 | 200 | 1090 |
| SVHN | ResN | ours | 0.755 | 6814 | 2530 | 0.577 | 1414 | 674 |
| | | top1 | 0.315 | 1665 | 142 | 0.267 | 452 | 122 |
| | | random | 0.343 | 3095 | 6249 | 0.086 | 210 | 1878 |
| | WRN | ours | 0.709 | 5062 | 2143 | 0.582 | 1404 | 1055 |
| | | top1 | 0.292 | 1238 | 130 | 0.203 | 275 | 85 |
| | | random | 0.28 | 2000 | 5205 | 0.095 | 229 | 2230 |
| | VGG | ours | 0.595 | 5214 | 3367 | 0.498 | 1272 | 911 |
| | | top1 | 0.172 | 840 | 67 | 0.139 | 221 | 52 |
| | | random | 0.341 | 2986 | 5595 | 0.094 | 240 | 1943 |
| F-MNIST | ResN | ours | 0.914 | 6034 | 873 | 0.791 | 2144 | 556 |
| | | top1 | 0.124 | 428 | 11 | 0.039 | 57 | 7 |
| | | random | 0.657 | 4340 | 2567 | 0.263 | 712 | 1988 |
| | WRN | ours | 0.896 | 5743 | 652 | 0.76 | 2033 | 641 |
| | | top1 | 0.144 | 490 | 14 | 0.045 | 63 | 8 |
| | | random | 0.638 | 4093 | 2302 | 0.281 | 752 | 1922 |
| | VGG | ours | 0.864 | 6348 | 1231 | 0.654 | 1895 | 1082 |
| | | top1 | 0.104 | 392 | 5 | 0.028 | 39 | 5 |
| | | random | 0.734 | 5393 | 2186 | 0.295 | 854 | 2123 |

Notice that DeepRobust-W's performance depends on the training data selection, mainly (a) how many weak vs. strong points are used to train the model, and (b) how many neighbors are generated per point to decide if it is strong/weak. To investigate (a), we assign a weight to each input point, indicating how likely it gets selected to train DeepRobust-W. In particular, for an input $i$, a weight $w_i := \frac{1+(1-n_i)^m \times 100^m}{1+100^m}$ is computed, where $n$ is its neighbor accuracy, and $m$ is a configurable parameter; with larger $m$, more weak points are sampled and DeepRobust-W will be trained with more weak points, and vice versa.

Table 5A shows the performance: as $m$ increases, the detector trades precision for recall. In this way, choosing different values of $m$, the precision-recall trade-off of the detector can be adjusted according to a user's need. From a different perspective, this way of oversampling weak points also addresses the potential problem of imbalanced data when the weak points are much less than the strong points.

Table 5: **DeepRobust-W performance using different sampling strategies for training**

**A: with varying number of strong/weak points**

| dataset | m | prec | recall | tp | fp | f1 |
|---|---|---|---|---|---|---|
| CIFAR-10 | 0 | **0.660** | 0.518 | 1290 | 664 | 0.581 |
| | 1 | 0.615 | 0.599 | 1490 | 932 | 0.607 |
| | 2 | 0.544 | **0.699** | 1740 | 1460 | **0.612** |
| SVHN | 0 | **0.677** | 0.502 | 1414 | 674 | 0.577 |
| | 1 | 0.575 | 0.653 | 1837 | 1357 | **0.612** |
| | 2 | 0.332 | **0.767** | 2160 | 4356 | 0.463 |
| F-MNIST | 0 | **0.794** | 0.787 | 2144 | 556 | **0.791** |
| | 1 | 0.746 | 0.839 | 2284 | 777 | 0.79 |
| | 2 | 0.712 | **0.871** | 2372 | 962 | 0.783 |

**B: with varying number of neigbours**

| dataset | #neighbors | prec | recall | tp | fp | f1 |
|---|---|---|---|---|---|---|
| CIFAR-10 | 6 | 0.662 | 0.389 | 967 | 493 | 0.49 |
| | 12 | 0.685 | 0.384 | 955 | 440 | 0.492 |
| | 25 | 0.665 | 0.502 | 1250 | 629 | 0.572 |
| | 50 | 0.660 | 0.518 | 1290 | 664 | 0.581 |
| | 200 | 0.683 | 0.507 | 1261 | 585 | 0.582 |
| SVHN | 6 | 0.723 | 0.403 | 1136 | 436 | 0.518 |
| | 12 | 0.672 | 0.527 | 1483 | 725 | 0.59 |
| | 25 | 0.619 | 0.629 | 1771 | 1090 | 0.624 |
| | 50 | 0.632 | 0.605 | 1703 | 993 | 0.618 |
| | 200 | 0.667 | 0.550 | 1550 | 774 | 0.603 |
| F-MNIST | 6 | 0.817 | 0.727 | 1981 | 443 | 0.77 |
| | 12 | 0.784 | 0.790 | 2153 | 592 | 0.787 |
| | 25 | 0.773 | 0.787 | 2143 | 629 | 0.78 |
| | 50 | 0.836 | 0.727 | 1981 | 390 | 0.778 |
| | 200 | 0.778 | 0.812 | 2211 | 632 | 0.794 |

Next, we check how DeepRobust-W's performance is dependent on the number of sampled neighbors, because a data point can potentially have infinite neighbors. Table 5B shows that the number of neighbors does not have much influence on the performance of the detector once it goes beyond some value (F1 score change less than 3.5 percentage point between 25 and 200 samples) for all the three datasets. Thus, we choose 50 for all of our experiments. For future work, a statistical bound with confidence intervals for neighbor accuracy can be estimated by modeling neighbor accuracy using distributions like folded normal.

**Result 2:** DeepRobust-W *can identify weak points with reasonably high F1 score: on average* 76.9%*, at* 0.75 *neighbor accuracy cut-off.*

## RQ3. Can we identify the weak points in a black-box setting?

We explore this RQ using DeepRobust-B, as discussed in Section 3.3. We assume only having access to unlabeled testing data and the model under test as a black-box. To evaluate DeepRobust-B, we spatially transform each test input $m$ times by randomly applying $d\omega \in [-30, +30]$ degrees rotation, $dx \in [-3, +3]$ pixels horizontal translation, and $dy \in [-3, +3]$ pixels vertical translation. We then calculate the output diversity score ($\lambda$) based on Equation (1) and rank the test images based on $\lambda$. Finally, we mark top $k$ images as potential most non-robust points. The parameter $k$ is chosen according to users' need.

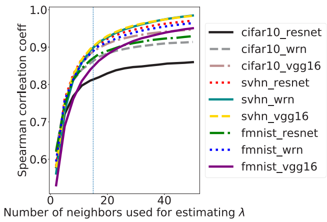

Fig. 6: **The spearman correlation coeff. between diversity score ($\lambda$) and neighbor accuracy, with varying #neighbors ($m$).**

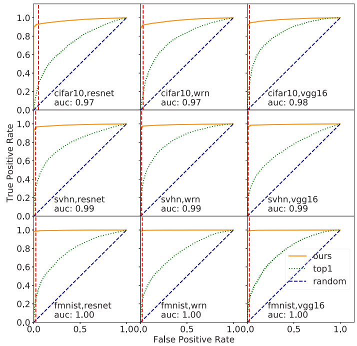

Fig. 7: **AUC-ROC curve with neighbor accuracy cutoff at 0.75. The red vertical line indicates when the diversity score threshold is chosen from training data.**

With each test data, DeepRobust-B queries the model with $m$ neighbors to compute $\lambda$. Since querying the classifier comes with an overhead, our goal is to achieve an optimal accuracy with minimal queries (i.e., $m$). To determine an optimal $m$ value, we explore the spearman correlation between diversity score and neighbor accuracy, with varying $m$, when running ResN on all the three datasets (see Figure 6). The correlation increases as $m$ increases, as with more query $\lambda$ becomes more accurate, and so the neighbor accuracy. We notice that at $m = 15$, the correlation coefficients across all the experimental settings reach above 0.8, and the rate of increase begins to slow down significantly. The results for the other two architectures are highly similar. Thus, we set $m = 15$ as default for DeepRobust-B.

Next, we evaluate DeepRobust-B's performance. We plot AUC-ROC by changing $top - k$ at $m = 15$ and compare our method with the random baseline and the top1 baseline as before. As shown in Figure 7, our method performs much better than the random baseline. In particular, our proposed method achieves AUC higher than 0.87 for all settings when neighbor accuracy cutoff is 0.5 and 0.97 when neighbor accuracy cutoff is 0.75.

Instead of above ranking based scheme, DeepRobust-B can also be used as a classifier if a diversity threshold is given (see Section 3.3). Here, we estimate the threshold using pre-annotated training data.

Table 6: **Performance of DeepRobust-B and the baseline methods for predicting weak points.**

| dataset | model | method | 75% | | | 50% | | |
|---|---|---|---|---|---|---|---|---|
| | | | f1 | tp | fp | f1 | tp | fp |
| CIFAR-10 | ResN | ours | 0.939 | 4714 | 257 | 0.622 | 1454 | 801 |
| | | top1 | 0.376 | 1218 | 206 | 0.182 | 255 | 120 |
| | | random | 0.501 | 2516 | 2455 | 0.234 | 549 | 1706 |
| | WRN | ours | 0.938 | 3657 | 171 | 0.585 | 986 | 604 |
| | | top1 | 0.35 | 889 | 222 | 0.183 | 189 | 90 |
| | | random | 0.383 | 1494 | 2334 | 0.182 | 307 | 1283 |
| | VGG | ours | 0.945 | 3397 | 148 | 0.682 | 1087 | 390 |
| | | top1 | 0.439 | 1070 | 153 | 0.266 | 278 | 106 |
| | | random | 0.36 | 1296 | 2249 | 0.153 | 244 | 1233 |
| SVHN | ResN | ours | 0.956 | 8371 | 365 | 0.67 | 1845 | 858 |
| | | top1 | 0.315 | 1665 | 142 | 0.267 | 452 | 122 |
| | | random | 0.336 | 2944 | 5792 | 0.102 | 280 | 2423 |
| | WRN | ours | 0.963 | 6827 | 227 | 0.718 | 1602 | 514 |
| | | top1 | 0.292 | 1238 | 130 | 0.203 | 275 | 85 |
| | | random | 0.275 | 1950 | 5104 | 0.085 | 191 | 1925 |
| | VGG | ours | 0.976 | 8608 | 144 | 0.779 | 2138 | 454 |
| | | top1 | 0.172 | 840 | 67 | 0.139 | 221 | 52 |
| | | random | 0.339 | 2997 | 5755 | 0.102 | 279 | 2313 |
| F-MNIST | ResN | ours | 0.987 | 6422 | 81 | 0.802 | 2316 | 546 |
| | | top1 | 0.124 | 428 | 11 | 0.039 | 57 | 7 |
| | | random | 0.655 | 4265 | 2238 | 0.289 | 835 | 2027 |
| | WRN | ours | 0.989 | 6246 | 70 | 0.857 | 2297 | 360 |
| | | top1 | 0.144 | 490 | 14 | 0.045 | 63 | 8 |
| | | random | 0.631 | 3987 | 2329 | 0.274 | 736 | 1921 |
| | VGG | ours | 0.991 | 7078 | 60 | 0.847 | 2393 | 418 |
| | | top1 | 0.104 | 392 | 5 | 0.028 | 39 | 5 |
| | | random | 0.711 | 5084 | 2054 | 0.277 | 784 | 2027 |

We evaluate precision and recall of DeepRobust-B in the nine DNN-dataset combinations under neighbor accuracy cutoffs 0.5 and 0.75. Table 6 shows the result. At 0.75 setting, DeepRobust-B has f1 up to 99.1%, with an average of 96.5%. At 0.50 setting, DeepRobust-B detects weak points with average f1 of 72.9%, while it can go up to 85.7%. It consistently produces better estimation than the top1 baseline and the random baseline. This shows that our black-box method can effectively identify weak points.

Note that, generating the spatial transformations and querying the model with it under black box setting is fast. Previous black box methods for adversarial perturbation work in such fashion [26,51]. For example, using CIFAR-10 , when we use a batch with size 100, the average transformation+query time for one image is $0.031 \pm 0.015$ ms. For the other two datasets, the overhead is similar. Thus, to for $m = 15$ queries, it takes only $0.465 \pm 0.225$ ms, which is a negligible overhead for most real-world DNN based vision applications. This implies that our black-box method can also be used in real time for many applications.

**Result 3:** *Given only black-box access to the DNN classifier,* DeepRobust-B *can identify weak points with f1 that are much better than those of using top1 method or random method.*

## RQ4. How generalizable are these findings?

The local robustness issues also exist in more critical applications like self-driving-car. Here we explore more complex transformations, i.e., adding rain and fog to the driving scenes. As shown in Figure 8, among those correctly classified data points, there is a non-trivial portion (45.8%) of them (in the heatmap, more red signified weaker) suffer from low (<0.75) neighbor accuracy.

Note that, here, we test regression models, which take images of driving scenes as inputs and output the corresponding steering angles.

Let a set of outputs predicted by a DNN be denoted by $\{\hat{\theta}_{o1}, \hat{\theta}_{o2}, ..., \hat{\theta}_{on}\}$, and ground truth labels for the original (unmodified) image points be $\{\theta_1, \theta_2, ..., \theta_n\}$. If the difference between predicted steering angle $\hat{\theta}_{oi}$ of a transformed image and the ground truth label of the original image $\theta_i$ is above a threshold, we consider it as incorrect.

The threshold $\lambda MSE_{orig}$ is defined following DeepTest's [73] as $MSE_{orig} = \frac{1}{n}\sum_{i=1}^{n}(\theta_i - \hat{\theta}_{oi})^2$ . $MSE$ is the Mean Square Error between the outputs and the manual labels, and $\lambda$ is a positive coefficient that is chosen to reflect a user's tolerance on the deviation. Note that there is no softmax layer (and thus no confidence score) in these regression models so the top1 baseline method cannot be used here.

Table 7 shows the result when $\lambda = 3$. At 0.75 setting, DeepRobust-W has f1 score up to 78.9%, with an average of 58.2%. At 0.50 setting, DeepRobust-W detects weak points with an average f1 of 47.9%, while it can go up to 68.2%. It consistently produces better estimation than the random baseline under all the settings. It should be noted that our observation is valid for all the $\lambda$ used in [73] from $\lambda$ equal to 1 to 5. This shows that our proposed method DeepRobust-W can be applied to regression problems with more complex natural transformations.

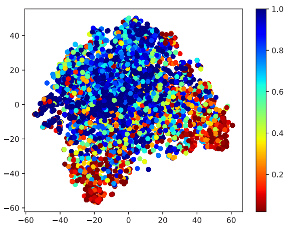

Fig. 8: **The t-SNE plot of correctly classified data points from Self-Driving dataset by the epoch model. data points are colored based on neighbor accuracy.**

| model | method | 0.75 neighbor acc. | | | 0.50 neighbor acc. | | |
|---|---|---|---|---|---|---|---|
| | | f1 | tp | fp | f1 | tp | fp |
| chauffeur | ours | 0.417 | 555 | 547 | 0.346 | 339 | 384 |
| | random | 0.146 | 194 | 908 | 0.096 | 94 | 629 |
| epoch | ours | 0.789 | 4354 | 1112 | 0.682 | 2641 | 1127 |
| | random | 0.586 | 3234 | 2232 | 0.411 | 1592 | 2176 |
| dave2 | ours | 0.541 | 979 | 471 | 0.409 | 475 | 246 |
| | random | 0.193 | 350 | 1100 | 0.121 | 141 | 580 |

Table 7: **Performance of DeepRobust-W for predicting weak points of Self-Driving dataset**

It should also be noted that it is unrealistic to use DeepRobust-B for this task for two reasons: It is impractical to try different variations of an image in real-time for a self-driving car, which is a time-sensitive application. Further, DeepRobust-B requires the calculation of neighbor diversity score. For a regression problem, the predicted values are continuous, so there is a very low probability for any two predictions being equal. Thus, the neighbor diversity score for every data point will be the same and cannot be used for identifying the weak points.

**Result 4:** DeepRobust-W *can detect weak points of a self-driving car dataset with f1 score up to 78.9%, with an average of 58.2%, at neighbor accuracy cutoff 0.75.*

## 6 Related Work

**Adversarial examples.** Many works focus on generating adversarial examples to fool the DNNs and evaluate their robustness using pixel-based perturbation [9, 17, 23, 25, 31, 36, 48, 49, 54, 63, 80–83]. Some other papers [14, 15, 86], like us, proposed more realistic transformations to generate adversarial examples. In particular, Engstrom et al. [14] proposed that a simple rotation and translation can fool a DNN based classifier, and spatial adversarial robustness is orthogonal to $l_p$-bounded adversarial robustness. However, all these works estimate the overall robustness of a DNN based on its aggregated behavior across many data points. In contrast, we analyze the robustness of individual data points under natural variations and propose methods to detect weak/strong points automatically.

**DNN testing.** Many researchers [16, 21, 29, 36, 41, 55, 69, 70, 74, 94] proposed techniques to test DNN. For example, Pei et al. [55] proposed an image transformation based differential testing framework, which can detect erroneous behavior by comparing the outputs of an input image across multiple DNNs. Ferit et al. [16] used fault localization methods to identify suspicious neurons and leveraged those to generate adversarial test cases.

In contrast, others [8, 29, 64, 73, 78, 92, 94] used metamorphic testing where the assumption is the outputs of an original and its transformed image will be the same under natural transformations. Among them, some use a uncertainty measure to quantify some types of non-robustness of an input for prioritizing samples for testing / retraining [8] or generating test cases[78]. We follow a similar metamorphic property while estimating neighbor accuracy and our proposed DeepRobust-B also leverages an uncertainty measure. The key differences are: First, we focus on estimating model's performance on general natural variants of an input rather than the input itself or only spatial variants. Second, we focus on the task of weak points detection rather than prioritizing / generating test cases. We also give detailed analyses of the properties of natural variants and propose a feature vector based white-box detection method DeepRobust-W. Further, we show that our method works across domains (both image classification and self-driving car controllers) and tasks (both classification and regression). Other uncertainty work complement ours in the sense that we can easily leverage weak points identified by DeepRobust-W and DeepRobust-B to prioritize test cases or generate more adversarial cases of natural variants.

Another line of work [18, 19, 27, 33, 34, 58, 72] estimates the confidence of a DNN's output. For example, [19] leverages thrown away information from existing models to measure confidence; [27] shows other NN properties like depth, width, weight decay, and batch normalization are important factors influencing prediction confidence. Although such methods can provide a confidence measure per input or its adversarial variants, they do not check its natural robustness property, i.e., with natural variations how will they behave.

**DNN verification.** There also exist work on verifying properties for a DNN model [7, 12, 24, 30, 56, 62, 83]. Most of them focus on verifying properties on $l_p$ norm bounded input space. Recently, Balunovic et al.[4] provides the first verification technique for verifying a data point's robustness against spatial transformation. However, their technique suffers from scalability issues.

**Robust training.** Regular neural network training involves the optimization of the loss for each data point. Robust training of neural network works on minimizing the largest loss within a bounded region usually using adversarial examples [15,35,43,45,50,75,81,83,84]. While both robust training methods and our work generate variants of data points, instead of training a model with these variants to improve robustness, we use them to estimate the robustness of unseen data points. The relation between robust retraining and our work is similar to bug fixing vs. bug detection in traditional software engineering literature.

## 7 Threats to Validity

We adopt rotation and translation as transformations for image classification tasks and rain and fog effects for the self-driving car task. There are many more natural variations such as brightness, snow effect etc. However, rotation and translation are representative of spatial transformation and used by many paper in evaluating robustness of DNN models[14, 55]. Rain and fog effects are also widely leveraged in many influential studies on testing self-driving cars [55,73,92].

Besides, for some of the experiments we did not show all the combinations under both neighbor accuracy cutoffs (i.e. 0.5 and 0.75). However, we note that the observations are consistent and we did not include them purely because of space limitation. Another limitation is that for both DeepRobust-W and DeepRobust-B, we need to decide the number of neighbors to use for training a classifier and estimating $\lambda$, respectively. We mitigate it by selecting the neighbor numbers that give stable performance in terms of precision and recall.

## 8 Conclusion and Future Work

In this work, we involve the data characteristic into the robustness testing of DNN models. We adopt the concept of neighbor accuracy as a measure for local robustness of a data point on a given model. We explore the properties of neighbor accuracy and find that weak points are often located towards corresponding class boundaries and their transformed versions tend to be predicted to be more diverse classes. Leveraging these observations, we propose a white-box method and a black-box method to identify weak/strong points to warn a user about potential weakness in the given trained model in real-time. We design, implement and evaluate our proposed framework, DeepRobust-W and DeepRobust-B, on three image recognition datasets and one self-driving car dataset (for DeepRobust-W only) with three models for each. The results show that they can effectively identify weak/strong points with high precision and recall.

For future work, other consistency analysis methods [18] e.g. variation ratio, entropy can be tried. We can potentially attain statistical guarantee for our black-box method by modeling the neighbor accuracy distribution and assume certain level of correlation between neighbor accuracy and complexity score. Besides, other definitions of robustness like consistency can be explored. We can also leverage ideas from [8,78] to easily prioritize test cases or generate more hard test cases based on identified weak points. Further, we can potentially modify existing fixing methods such as [20] targeting the weak points to fix them.

## 9 Acknowledgement

We thank Mukul Prasad and Ripon Saha from Fujisu US for valuable discussions. This work is supported in part by NSF CCF-1845893 and CCF-1822965.
